# Enteric Tuft Cells in Host-Parasite Interactions

**DOI:** 10.3390/pathogens10091163

**Published:** 2021-09-09

**Authors:** Sruthi Rajeev, Olivia Sosnowski, Shuhua Li, Thibault Allain, André G. Buret, Derek M. McKay

**Affiliations:** 1 Gastrointestinal Research Group, Department of Physiology and Pharmacology, Calvin, Phoebe and Joan Snyder Institute for Chronic Diseases, Cumming School of Medicine, University of Calgary, Calgary, AB T2N 4N1, Canada; sruthi.rajeev1@ucalgary.ca (S.R.); shuhua.li@ucalgary.ca (S.L.); 2 Inflammation Research Network and Host-Parasite Interaction Group, University of Calgary, Calgary, AB T2N 4N1, Canada; olivia.sosnowski@ucalgary.ca (O.S.); thibault.allain@ucalgary.ca (T.A.); aburet@ucalgary.ca (A.G.B.); 3 Department of Biological Sciences, Faculty of Science, University of Calgary, Calgary, AB T2N 1N4, Canada

**Keywords:** epithelial chemosensors, Th2 effector, gastrointestinal disorders, helminths, protozoa, coinfections

## Abstract

Enteric tuft cells are chemosensory epithelial cells gaining attention in the field of host-parasite interactions. Expressing a repertoire of chemosensing receptors and mediators, these cells have the potential to detect lumen-dwelling helminth and protozoan parasites and coordinate epithelial, immune, and neuronal cell defenses against them. This review highlights the versatility of enteric tuft cells and sub-types thereof, showcasing nuances of tuft cell responses to different parasites, with a focus on helminths reflecting the current state of the field. The role of enteric tuft cells in irritable bowel syndrome, inflammatory bowel disease and intestinal viral infection is assessed in the context of concomitant infection with parasites. Finally, the review presents pertinent questions germane to understanding the enteric tuft cell and its role in enteric parasitic infections. There is much to be done to fully elucidate the response of this intriguing cell type to parasitic-infection and there is negligible data on the biology of the human enteric tuft cell—a glaring gap in knowledge that must be filled.

## 1. Introduction

Parasitic helminths and protozoa that infect the intestine have a range of deleterious effects in humans, companion animals and domestic livestock across the globe [1,2,3]. Laboratory models (predominantly rodent) of parasitic infections are valuable in assessing the host-parasite interaction and in identifying potential therapeutics. In response to the parasite, either free in the gut lumen or attached to/embedded in the epithelium, the host seeks to mount a coordinated multicellular response executed by epithelial, stromal and immune cells, nerves and muscles in order to inactivate and/or expel the parasite from the body [4,5]. The enteric tuft cell (ETC), an epithelial cell type present in the small and large intestines, has recently attracted attention for its role in coordinating anti-parasite immunity following infection with helminth and protozoan parasites [6,7,8,9,10]. Although ETCs were described almost 70 years ago [11], the literature describing their role in parasitic infections, inflammatory diseases and viral pathogenesis is at a nascent stage. We review the features that place ETCs at a potentially critical nexus in host-parasite interactions: (i) exploring the crosstalk between ETC-derived mediators with other cell types coordinating the host immune responses; (ii) describing the range of tuft cell sensing during parasitic infections; and, (iii) exploring the potential links between ETCs, gastrointestinal diseases and enteric parasites. By providing insight and critique we hope to prompt research towards unexplored territories in the burgeoning field of ETC sensing and responding to parasites in the mammalian intestine.

## 2. Tuft Cell Lineage, Subtypes, and Basic Biology

Tuft cells, commonly referred to in early studies as brush cells, are aptly named for their characteristic tuft-shaped apical microvilli [12,13]. They occupy the epithelial compartments of multiple organs including the airways, lungs, thymus, stomach, small and large intestines, pancreas and urethra [14,15,16,17,18,19,20]. Following three seminal publications in 2016, there has been a rapid expansion of interest in the enteric tuft cell and its contribution to gut homeostasis and the response to infection [6,7,9]. The majority of knowledge of the ETC has been gleaned from analyses in mice. Gene expression analyses via single cell RNA sequencing and gene microarrays have placed tuft cells among chemosensory cell types, characterized by the expression of receptors for taste ligands, parasite and/or bacteria-derived metabolites, as well as host immune and neuronal mediators [21,22].

The total tuft cell population increases post-weaning in mice [23], yet ETCs are sparse in adult mice representing ~0.5% of the epithelial cell population in the small intestine and colon at steady state (reported range = 0.4–2%) [9,24,25]. Data in favor of a proximal-to-distal gradient and, conversely, a distal-to-proximal gradient in murine ETC numbers have been reported, reflective of the not unexpected contradictory findings in a new area of research [25,26]. The small intestinal tuft cell population expands dramatically during parasitic infections [9].

Like all gut epithelial cells, ETCs arise from the intestinal crypt stem cells (ISCs) and their differentiation is dependent on the transcription factors POU Domain Class 2 Transcription Factor 3 (POU2F3) and Growth Factor Independent 1b (Gfi1b) [9,27]. There are contrasting data on the dependence of intestinal tuft cell differentiation on atonal homolog 1 (ATOH1), a transcription factor involved in the differentiation of goblet, Paneth and enteroendocrine cells: all secretory cell types [28]. In the small intestine, depleting ATOH1 expression in intestinal epithelial stem cells has been reported to result in tuft cell hyperplasia using *Lrig1Cre^ERT2/þ^; Atoh1^fl/fl^* mice [13,27,29,30,31] contradicting the conclusions drawn by reports showing small intestinal tuft cell depletion in *Villin-Cre^ERT2^Atoh^fl/fl^* mice—where ATOH1 expression is depleted in all small intestinal epithelial cells [31,32]. While small intestinal organoids derived from *Villin-Cre^ERT2^Atoh^fl/fl^* mice are devoid of tuft cells at homeostasis, IL-13 induces tuft cell hyperplasia, suggesting that small intestinal tuft cell differentiation in response to this Th2 cytokine is ATOH1-independent [31]. In response to enteric parasites that colonize the small intestine, increased IL-13 production by immune cells induced stem cells to differentiate to the tuft and goblet cell types via IL-4Rα signaling in the small intestine [6,9,33], but not in the colon [6]. Interestingly, differentiation of colonic tuft cells was found to be ATOH-1 dependent [13,30,34] and colonic tuft cell hyperplasia is recorded in response to changes in the microbiome and during acute inflammation [25,35]. Such variation in the regulation of differentiation hints at the probability that small intestinal and colonic ETCs may perform different functions [30].

ETCs share many similarities with taste cells. For example, POU2F3 is required for type 2 taste cell differentiation in the taste buds of the tongue [36] and ETCs and taste cells express taste transduction pathway components including transient receptor potential cation channel subfamily M member 5 (TRPM5) and α-gustducin [21] (see below). Similar to taste cells, ETC activation following infection with parasitic helminths is dependent on TRPM5: the cation channel, which aids in the depolarization of the cell (Ca^2+^ ion activated and allowing influx of Na^+^ ions) and exocytosis of IL-25 [7,8,23].

### Tuft Cell Subtypes and Specific Functions

Transcriptome analysis has been used to propose grouping ETCs into tuft 1 (neuronal) and tuft 2 (immune) cell subtypes. Tuft 1 and tuft 2 cells both express doublecortin-like kinase 1 (*Dclk1*) and *Il-25*, genes encoding for a microtubule-associated kinase and an alarmin cytokine, respectively [22]. Tuft 1 cells express higher levels of neuronal genes such as *Nradd, Nrep* and *Ninj1* whereas tuft 2 cells express higher levels of immune genes including those that code for thymic stromal lymphopoietin (TSLP) and CD45 [22]. Closer analysis suggests that tuft 1 cells represent the majority of ETCs at baseline conditions, while tuft 2 cells expand in number in the mouse small intestine following infection with the parasitic nematode *Heligomosomoides polygyrus* [22]. Three-dimensional organoid cultures were shown to lose DCLK1^+^ tuft cell numbers over a week of culture unless they were co-cultured with primary neurons or supplemented with the cholinergic agonist pilocarpine, suggesting a critical role for neuronal mediators in the maintenance of tuft cells in culture [37]. Recently, treatment with scopolamine, a muscarinic cholinergic receptor antagonist, resulted in the induction of another subset of ETCs with a genetic signature resembling enteroendocrine cells and distinct from the previously described tuft 1 and 2 subtypes [38]; the identity of this third type of ETC awaits validation in other conditions. Whether these subtypes of tuft cells arise from a common precursor or if a single cell can switch between phenotypic characteristics is unclear, as is the cell-specific bioactivity of the ETC types. For instance, while hyperplasia of small intestinal DCLK1*^+^* ETCs is a reproducible finding following infection of mice with a helminth parasite, whether this population is of a single phenotype, or a mixture of tuft subtypes remains unknown.

In other endoderm-derived organs, tuft cells share similar gene expression patterns, yet they may perform specific roles that cater to their niche: thymic tuft cells may be involved in educating T cells and influencing the development of Natural Killer T cells and B cells [39,40]; tracheal tuft cells coordinate mucociliary clearance responses to bacterial peptides [41]; pancreatic tuft cells have been described in mouse models of ductal metaplasia where their production of prostaglandins suppresses tumorigenesis [18,42]. These examples of organ- or tissue-specific roles of tuft cells support the postulate that ETC phenotype and activity is context-dependent and may also vary based on the parasitological stimulus.

## 3. Host-Parasite Interactions: The Role of Enteric Tuft Cells

Parasitic helminths can reside in the lumen of the gut or invade the epithelium, lamina propria or muscle layers of the intestine, depending on their life cycle and tissue tropism (Table 1) [4,43,44,45,46,47,48,49]. In most cases, host resistance to helminth-infection is characterized by a Th2 immune response as seen with the nematodes *Nippostrongylus brasiliensis*, *Trichinella spiralis* and *H. polygyrus* (Table 1) [4,45,48,50]. Helminths also induce regulatory anti-inflammatory cytokines (e.g., IL-10), regulatory T and B cells and macrophages to suppress tissue inflammation [51], revealing a bidirectional dialogue between host and parasite. On the other hand, the trematode *Echinostoma caproni* and the nematode *Trichuris muris* which elicit Th1 responses, in certain mouse strains, are likely to manifest as chronic infections in these hosts [52,53,54,55].

**Figure 1:**
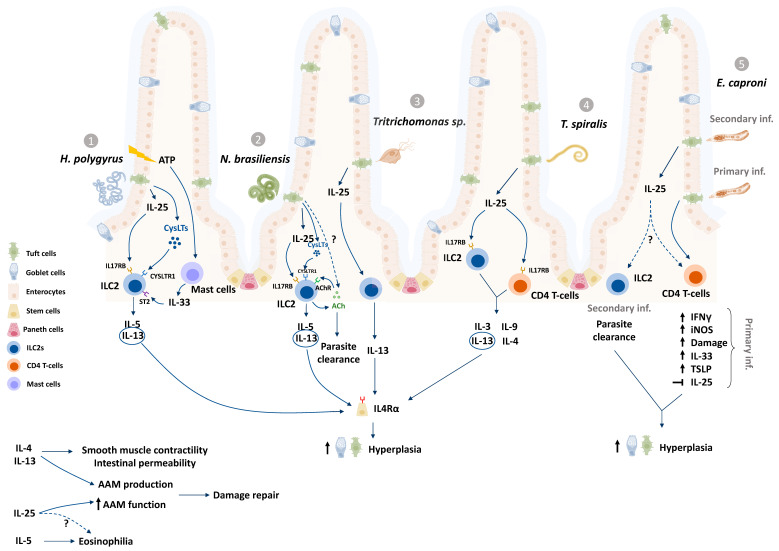
Enteric tuft cells (ETCs) have been studied in murine infection models with the nematodes *Heligosmoides polygyrus* (1), *Nippostrongylus brasiliensis* (2), and *Trichinella spiralis* (4); the trematode *Echinistoma caproni* (5), and the protozoan *Tritrichomonas (3).* In response to helminths and protozoans, ETCs produce and release IL-25 (1-4) and cysteinyl leukotrienes (1,2), which subsequently activate the innate lymphoid cell (ILC2) population in the underlying lamina propria via IL-17RB and CYSLTR receptors to produce IL-13, IL-5 and IL-9 [6,7,9,57,64]. IL-13 induces intestinal stem cell differentiation via IL-4R**α** signaling, causing ETC and goblet cell hyperplasia (see Figure 2 for approximate timelines), increased smooth muscle contractility and intestinal permeability amongst other mechanisms that aid parasite expulsion [5,9]. Mast cells sense damage related release of ATP and release IL-33 to activate ILC2 production of IL-13 (1) [43]. ILC2s also produce and release acetylcholine (ACh) in response to infection with *N. brasileinsis* and are activated by ACh to produce IL-13 (2) [65,66]. Although ETC hyperplasia is observed after primary and secondary infection with *E. caproni*, *Il-25* expression is upregulated in the acute secondary infection rather than the chronic primary infection, which is characterized by increased levels of Th1 cytokine mRNA (5) [10,53,63]. ETC-derived mediators may also act on other cell types such as eosinophils and macrophages (alternatively activated macrophages, AAM), to coordinate repair, as well activate CD4 T cell populations (4,5) to release cytokines during parasitic infections [48,67,68,69].“?” represents mechanisms that are yet unknown in tuft cell literature.

Increasing evidence (Table 1, Figure 1) suggests that ETCs play major roles in the development of a Th2-dominated host immune response to helminths as well as a murine protozoan, *Tritrichomonas* [6,7,8,9,23,56,57,58]. *Tritrichomonas muris* chronically colonizes the lumen of the distal small intestine and large intestine of mice and elicits colonic Th1 and Th17 cytokine responses as well as an expansion of type 2 innate lymphoid cells (ILC2s) [7,56,59,60]. Human protozoan parasites such as *Cryptosporidium* species*, Entamoeba histolytica* and *Giardia lamblia* are major causes for food and waterborne human diarrhea globally of [1]. Whether ETCs respond to the protozoans *Cryptosporidium* and *Entamoeba* remains to be tested even though host resistance in both cases are characterized by Th1 dominant responses [61,62]. Recent discoveries also encourage discussion around ETC functions beyond mobilization of anti-worm responses, such as tissue repair and protection from secondary infections [6,7,9,10,50,57,58,63], and provide additional rationale to study ETC responses to enteric parasites that infect humans and livestock.

**Table 1:** Murine parasitic infections that engage ETC activity ^1^.

Parasite	Region of Infection	Migration/Invasiveness of Parasite	Host Immune Response	Type of Infection	Receptors Implicated in ETC Activation	TRPM5 → ETC Hyperplasia?	Evidence of ETC Mediator Post Infection
**Helminths**							
**Nematodes**							
*Nippostrongylus brasiliensis*	Lung, SI	Migratory: skin → lungs → GI tract [45]	Th2	Acute	Not via SUCNR1 [56]	Yes [56]	IL-25: Yes [6,9] CysLT: Yes [57]
*Heligosmoides polygyrus*	SI: duodenum, jejunum	Non-migratory Invasive: invades intestinal epithelium [50]	Th2	Chronic	Not via TAS1R3 [58]	Yes [6,62]	IL-25: Yes [63] CysLT: Yes [57]
*Trichinella spiralis*	SI: jejunum	Migratory: stomach → intestine → lymphatics → muscles [48,49]	Th2	Acute (intestine) Chronic (muscle)	TAS2R (tested ex vivo) [8]	Yes [8]	IL-25: Yes [8] CysLT: ?
**Trematodes**							
*Echinistoma caproni*	SI: ileum	Non-migratory Attaches to mucosa [55]	Th1 (1°) Th2 (2°)	Chronic (1°) Acute (2°)	?	?	*Il-25* (mRNA): No (1°), Yes (2°) [10,53,63] CysLT: ?
**Protozoan**							
*Tritrichomonas* spp.	SI: ileum; colon,cecum	Non-migratory Non-invasive [59,60]	Th2 (SI) Th1,Th17 (colon,cecum)	Chronic: commensal microbe	SUCNR1 (Tm, Tr) [23,56] TAS1R3 (baseline regulation of ETCs) (Tm) [58]	Yes [62]	*Il-25* (mRNA): Yes (Tm, Tr) [23,56] CysLT: No (T. mu) [57]

^1^ ETC = enteric tuft cell, TRPM5 = Transient receptor potential cation channel subfamily M member 5, IL-25 = interleukin 25, CysLT = cysteinyl leukotriene, SI = small intestine, SUCNR1 = succinate receptor, TAS1R3 = sweet/umami taste receptor subunit, TAS2R = bitter taste receptor, 1° = primary infection, 2° = secondary infection, “?”= no data found, Tm = *Tritrichomonas muris,* Tr = *Tritrichomonas rainier*, T. mu = *Tritrichomonas musculis.*

### 3.1. Tuft Cell Contributions to the Th2 Response to Helminths

The Th2 response consists of a network of defences, characterized by the production of IL-4, -13, -9 and -5 (reviewed by [70]), the accumulation of eosinophils and macrophages, and the development of parasite-antigen specific T and B cells [4,5,71]. ILC2s, a group of lineage negative non-antigen specific cells, produce IL-13 upon activation by the alarmin cytokines IL-25 and IL-33 during infection with helminths [72]. ETCs are major producers of IL-25 in the intestine for homeostatic maintenance of tuft cells in a paracrine signaling loop [6]. ETC-derived IL-25 production has been observed in response to *N. brasiliensis*, *H. polygyrus*, *T. spiralis* and *Tritrichomonas muris* infections (Table 1, Figure 1) [6,7,8,9]. ETCs also produce and release cysteinyl leukotrienes (CysLTs), potent eicosanoid lipid mediators, in response to *N. brasiliensis* and *H. polygyrus* but not *Tritrichomonas musculis* [57]. Recently, murine ETCs were shown to activate the tumor suppressor gene *p53* following *N. brasileinsis* and *Tritrichomonas muris* infection [73]. One consequence of this *p53* activation was upregulated expression of the lymphoid-restricted membrane protein (LRMP1), which, in association with the channel protein ITPR2 (Inositol 1,4,5-Trisphosphate Receptor Type 2), coordinates the release of Ca^2+^ ions and subsequent release of IL-25 via TRPM5 [73]. This adds another level of intracellular control over IL-25 to regulate the activation of a Th2 cascade.

Both IL-25 and CysLTs are implicated in the activation of a tuft-ILC2 circuit, wherein ILC2s expressing the IL-17RB receptor subunit for IL-25, and the CysLT receptor are stimulated to release IL-13 [6,9,57]. IL-13 integrates the Th2 response by stimulating multiple Th2 effector cells including eosinophils, macrophages and B cells, and by driving goblet cell hyperplasia [74]. IL-13 (and IL-4) also coordinates the “weep and sweep” response, whereby the host secretes mucus and water into the lumen to trap and flush out the parasite (“weep” stage) by increased peristaltic contractions (“sweep” stage) from the intestine [5,6,9].

### 3.2. The Dynamic between Tuft Cells, Goblet Cells, and Mucus Defense

**Figure 2:**
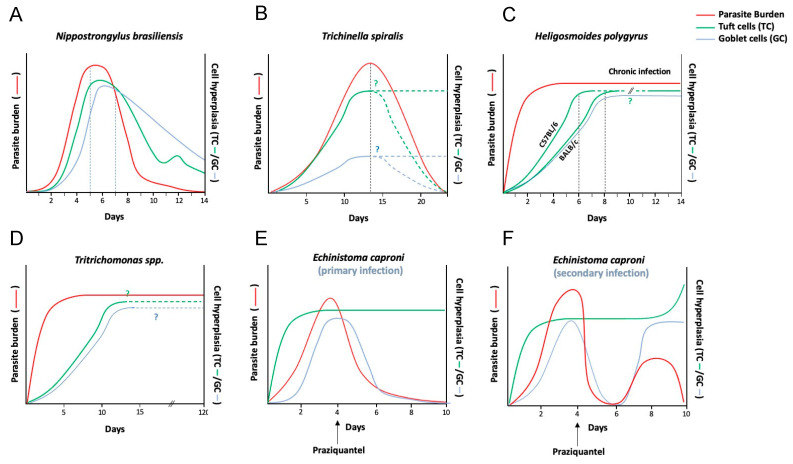
Dynamics of parasite clearance, tuft cell hyperplasia and goblet cell hyperplasia during parasitic infections. In some acute enteric infections, such as with (**A**,**B**) *N. brasiliensis* and *T. spiralis* (intestinal phase) the events of enteric tuft cell (ETC) hyperplasia and goblet cell hyperplasia precede or happen around the same time as worm expulsion/reduction in worm burdens [6,8,9,80,81]. In chronic parasitic infections however, such as with (**C**) *H. polygyrus* [6,9,46,82,83], (**D**) the protozoan *Tritrichomonas* [59] and (**E**) *E. caproni* [55], ETC hyperplasia persists for as long as parasite burden persists [7,10,23] and may also coincide with goblet cell hyperplasia [7,10,56]. It remains to be tested whether ETC hyperplasia (as well as goblet cell hyperplasia) serves to either induce immunity against secondary/concomitant infections or mediate repair of damaged intestinal tissue [23]. ETC hyperplasia in parasitic models of infection may be the downstream effect of a Th2 cascade (dominated by increased IL-13 production in the intestinal niche) [9] with the exception of the (**E**,**F**) *E. caproni* trematode model of murine infection, where primary host response is Th1 cytokine centric [53], and ETC hyperplasia is observed in both primary infection as well as secondary infection after drug clearance (praziquantel) [10]. Graphs represent hypothetical temporal kinetics of worm burden, tuft cell and goblet cell numbers based on individual time point data presented in literature, where “?” and dotted lines represent estimation of trendlines at time points where data was unavailable.

Goblet cells are a key component of the host defence against parasites, producing and releasing mucus to provide a layer of protection in the intestine [75,76]. The timelines of ETCs and goblet cell hyperplasia are coincident following infection with helminth parasites (Figure 2). Tuft cell deficient animals (*Pou2f3^−/−^)* show reduced total goblet cells and only a focal goblet cell hyperplasia, as well as reduced goblet cell expression of *Retnlβ* mRNA (codes for Resistin-like molecule (RELM) β) in response to infection with *N. brasiliensis* [9]. RELMβ is a mucus defensin shown to dampen nematode movement towards the epithelium [77]. Another way by which the ETC response to parasites may coordinate goblet cell responses is by releasing the secretagogues acetylcholine (ACh) and prostaglandins which can stimulate goblet cell release of mucus and increase mucus gel thickness, respectively [78,79]. Although tuft cells have been shown to release ACh in the airway and prostaglandin in the pancreas, it is uncertain whether ETCs can release either of these mediators [15,42].

### 3.3. ETCs May Mediate Concomitant Immunity from Secondary Parasitic Infections

In the *Pou2f3^−/−^* mouse, expulsion of *N. brasiliensis* is delayed [6,9], indicating that in this model of infection with helminth parasites, ETCs coordinate timely worm expulsion via the initiation of a type 2 immune response. The association between ETC hyperplasia and parasite eradication, however, is not always this clear. In addition, whether ETC numbers remain elevated throughout other chronic parasitic infection models as seen in *H. polygyrus*-infected mice (Figure 2) [23] has not been tested yet, prompting the question—do hyperplastic ETCs serve to perform functions beyond facilitating parasite clearance in primary infections? For instance, hyperplastic ETCs may facilitate protection from secondary parasitic infections.

*Tritrichomonas* colonization in mice with constitutive activation of the tuft-ILC2 circuit, due to uninhibited IL-25 activation of ILC2s (*A20^fl/fl^*), confers a measure of protection against infection with the helminths *H. polygyrus* and *N. brasiliensis,* as measured by reduced worm fecundity and worm burdens which was abrogated in tuft cell-deficient mice [23]. Likewise, mice that had previously cleared *N. brasiliensis* were resistant to *H. polygyrus* (which normally exhibits as a chronic infection) [23]. In *E. caproni*-infected mice, ETC hyperplasia was observed during a chronic, Th1-inducing primary infection, with no concurrent increase in the intestinal levels of IL-25 [10,53]. Upon secondary infection with *E. caproni* (i.e., post-drug clearance of the primary infection) both ETC hyperplasia as well as an increase in tissue IL-25 levels were observed alongside worm clearance (Figure 2) [10,63]. The mode by which IL-25 is stimulated during secondary protection remains unknown [10,63], however there was also increased expression of IL-13 [63].

These findings are consistent with the position that the activation of ETCs, and subsequently ETC hyperplasia, may exert protective effects against subsequent or concomitant parasitic infections. However, in order to confirm or refute this suggestion carefully controlled experiments with *Pou2f3*^−/−^ mice are required [23], as well as consideration of the mucosal immune response and perturbations to the gut microbiome in primary-secondary infections or coinfection paradigms. 

### 3.4. Alternatives to the “Tuft–ILC2 Circuit”

The tuft cell-ILC2 circuit has been explored in rodent-parasite model systems (Figure 1) as a key pathway that kickstarts host anti-parasitic responses [6,7,9,57]. While ILC2s are indispensable for the expulsion of *N. brasiliensis* from mice [72], they may play redundant roles in resistance to other helminths, as is evidenced with *H. polygyrus* [64]. Supplementing *Rag-1^−/−^* mice (which lack mature B and T cells) with recombinant IL-25 mediates reduction in *H. polygyrus* worm burdens, but in an ILC2-independent manner [64]. IL-25 supplementation was found to stimulate increased numbers of eosinophils and M2 macrophages in *H. polygyrus*-infected mice, which may have contributed to reduced worm burdens [64]. Macrophages, eosinophils and mast cell progenitors express the IL-17RB receptor subunit [64,67,84,85], and may therefore respond directly to ETC-derived IL-25 to contribute to anti-helminth responses and worm expulsion.

### 3.5. ETCs Contribute to Small Intestinal Remodeling and Epithelial Restitution upon Infection

Many parasites damage the intestinal mucosa due to larvae migration, adult attachment or colonization of the epithelium directly (Table 1). An increasing body of evidence suggests that tuft cells may coordinate the adaptive remodeling of the small intestine following parasitic infections in an IL-25-dependent mechanism [23]. ETC-derived IL-25 may also act on immune cells such as macrophages and eosinophils and aid in the healing of the small intestine following infection with helminth parasites.

Alternatively activated macrophages (AAMs) are a class of cells activated by IL-4/IL-13 (and a variety of other stimuli to give a spectrum of regulatory macrophages) that play important roles in mucosal wound healing [86,87]. In vitro experiments showed that bone marrow-derived murine macrophages treated with IL-4/IL-13 respond to IL-25 by increasing the expression of AAM associated genes, such as *Arg1*, *Ym1* and *Fizz1* [88], used by AAMs to coordinate collagen formation and myofibroblast differentiation among other key events in fibrosis and wound healing [89]. Macrophage inhibitory factor (MIF), a cytokine that activates and sustains reparative macrophages in helminth infections [90], mRNA expression is twofold higher in ETCs than in other intestinal epithelial cells [21], and may represent another way in which ETCs regulate the macrophage responses during helminth infections.

Eosinophilia is a common feature of infection with helminth parasites [71]. While excessive eosinophil degranulation and release of mediators can damage the worm, these cells are not indispensable for worm expulsion as shown by studies of helminth-infected eosinophil depleted/deficient mice [91,92,93]. Instead, eosinophils may maintain gut immune homeostasis by promoting the maintenance of IgA-producing plasma cells [94]. Eosinophils can produce cytokines such as transforming growth factor β (TGFβ) to induce tissue remodeling as seen in the airways [95]. In a murine *Clostridium difficile*-infection (bacterial) model, cecal production of IL-25 resulted in the recruitment of eosinophils [69]. IL-25 also induces eosinophil migration in allergic airway models of hypersensitivity [85], suggesting that ETC-derived IL-25 would contribute to eosinophilia following infection with helminth parasites.

Reports also indicate that tuft cell DCLK1 may aid tissue repair [96]. In a non-parasitic model of radiation-induced DNA damage, tuft cell expression of DCLK1 was found to be protective, and directly linked to expression of the ATM-mediated (ataxia telangiectasia mutated) damage repair pathways [96]. DCLK1 was also found to regulate mRNA expression of cyclooxygenase 2 (COX-2), an enzyme involved in ETC production of prostaglandins, which can in turn contribute to epithelial repair [21,22,97]. Interestingly, prostaglandins were found to suppress differentiation towards the tuft cell fate in colonic organoids, the effect being reversed when colonic organoids were treated with aspirin, a COX-1 and -2 inhibitor [98], suggesting that ETCs could potentially self-limit their numbers/functions by releasing prostaglandins.

### 3.6. Tuft-Cell-Derived Neuronal Mediators and Anti-Parasitic Responses

ETCs have been found to express genes that are typical of neurons, such as those encoding for presynaptic and postsynaptic proteins [21], choline acetyltransferase (ChAT) (the enzyme required for synthesis of ACh) [99], receptors for dopamine (Drd3), and gamma-aminobutyric acid (GABA) [21,22,100]. Immunohistochemical studies also suggest that ETCs are in close proximity to sensory neurons [21,99]; however, the specificity of the association was not tested and it is unclear if the degree of proximity identified would enhance bidirectional communication between nerve and tuft cell.

Tuft cells in the airways and urethra have been shown to release ACh to activate neurons and coordinate reflexes to pathogenic stimuli [19,41]. In the gut, ACh stimulates lumen-directed secretion of chloride by enterocytes as a driving force for water movement [101], coordinates smooth muscle contractility [102], acts on intestinal stem cells to induce differentiation [103] and induces mucus exocytosis from goblet cells [78]; events that all contribute to the anti-parasite response. Recently, ChAT^+^ ILC2s in the intestine and airways were shown to produce and release ACh in response to the helminth parasite *N. brasiliensis*, and perturbation of this ability resulted in an increased worm burden [65,66]. ACh-stimulated ILC2s display increased production of IL-13 and IL-5 in vitro [65], suggesting autocrine as well as paracrine positive feedback loops where ILC2-derived ACh as well as ETC-derived ACh may potentiate ILC2 responses.

While ETCs stain positive for ChAT-immunoreactivity [99], it is unknown whether they release ACh in response to parasitic infections. As noted, treatment with scopolamine, an anti-cholinergic drug, induced ETC hyperplasia, further suggesting a role for ACh signaling in tuft cell activity [38].

A subset of murine ETCs has been identified as 5-hydroxytryptamine (5-HT, serotonin) positive by immunostaining. Serotonin can promote the expulsion of enteric parasites by inducing water movement into the gut lumen and increasing peristalsis [104,105]. However, tryptophan hydroxylase (TPH-1), which is necessary for 5-HT production [21,104] has not been demonstrated in the ETC, casting doubt on the former data. As an immunomodulatory signal in the gut, it will be important to determine whether ETCs are a significant source of 5-HT (as compared to the enteroendocrine cell), and, if so, how ETC-derived 5-HT participates in the host-parasite interaction [106].

## 4. Tuft Cell-Parasite Communication: Which Parasitic Factor Induces the Tuft Cell Response?

Tuft cells may detect luminal and mucosal signals via a variety of apical and basolateral receptors. They express multiple elements of chemosensory machinery including (i) taste receptors (TAS2Rs and TAS1R3), (ii) components of the taste transduction pathway (G-protein associated subunit α-gustducin and TRPM5), and (iii) metabolite sensing receptors [8,21,23,58]. Additionally, ETCs express receptors for endogenous mediators such as IL-25, GABA and dopamine [22,100,107]. To fully comprehend tuft cell involvement in anti-parasitic responses, it is important to investigate the parasite-derived factors that activate ETC responses: an area of study also currently hampered by the lack of specific markers that indicate ETC activation. Parasite-specific stimuli include excretory/secretory metabolic products (ESPs) [8,57], exosomes/microvesicles and surface antigens present on the parasite [8,98,108]; all of which will vary with the life-cycle stage of the parasite. Additionally, host immune and/or neurone-derived molecules produced as a consequence of infection may have the capacity to affect ETC function, presenting the field with numerous possibilities that warrant future investigation.

### 4.1. “Tasting” Parasites and Tuft Cell Functions

Tuft cells express receptors that, on taste cells in the tongue, function to detect bitter, sweet and umami taste ligands. For instance, TAS2Rs are a group of G-protein coupled receptors (GPCRs) with multiple subtypes that respond to bitter taste ligands such as flavonoids, peptides, alkaloids, and denatonium [109]. There are 35 murine *Tas2r* genes, some of which share homology with human TAS2Rs [8]. Infection with the nematode *T. spiralis* results in upregulated gene expression of eight subtypes of bitter taste receptors (and the downregulation of eight other subtypes) in the murine intestinal epithelium [8]. *T. spiralis* ESPs and sonicated antigenic mixtures of *T. spiralis* induce primary intestinal epithelial cells (IECs) ex vivo to release IL-25 in a TRPM5-dependent manner, an effect inhibited by the bitter taste receptor antagonist AITC [8]. It is noteworthy that extracts from encysted larvae in the somatic muscle and adult lumen-dwelling *T. spiralis* activate the IEC release of IL-25 [8]. As ETCs are suggested to be the dominant epithelial source of IL-25, it is likely that IL-25 release upon *T. spiralis* infection is ETC derived; however, tuft cell specificity in the activation of the bitter taste receptor in this model of infection requires rigorous testing.

The taste receptor subunit TAS1R3 dimerizes with TAS1R2 or TAS1R1 to respond to sweet or umami taste ligands [109]. Murine ETCs express functional TAS1R3 receptors in a strain dependent manner wherein BALB/c mice, unlike C57BL/6j mice, have an inactive form of TAS1R3 [58]. *Tas1r3^−/−^* mice show reduced ETC numbers at baseline conditions, suggesting a role for this taste receptor subunit in regulating the number of ETCs during homeostasis, whereas *Tas1r1* and *Tas1r2* mRNAs are not detected in the small intestine of either strain [58]. While *Tas1r3^−/−^* mice show delayed ETC hyperplasia in response to succinate supplementation and infection with the protozoan *Tritrichomonas muris*, the magnitude of ETC hyperplasia in response to *H. polygyrus* was similar to wild-type mice indicating that the helminth-evoked response was not dependent on *Tas1r3* [7].

Tuft cells in other organ systems have also demonstrated the ability to respond to taste ligands. For example, urethral tuft cells activated by denatonium and monosodium glutamate (bitter and umami taste ligands) release ACh to stimulate the micturition reflex [19]. Tracheal tuft cells respond to both bitter taste ligands as well as quorum sensing molecules released by pathogenic biofilm forming *Pseuodomonas aeruginosa* to stimulate the mucociliary clearance response [16,17]. However, helminth-derived molecules that specifically bind to and activate tuft cells remain elusive. Knowledge of these molecules would, in theory, allow the design of orally available drugs that would drive Th2 immunity as a means to control Th1 dominated immunopathologies in the gut.

### 4.2. Tuft Cells Respond to Alterations in Microbial/Parasitic Metabolites

Tuft cells express the succinate receptor, SUCNR1 (GPR91) [21]. Commonly produced by bacteria as well as by certain parasites, including by *Tritrichomonas* and *N. brasiliensis*, succinate induces ETC release of IL-25 with a subsequent downstream effect being ETC hyperplasia presumably via ILC2 released IL-13 acting on stem cells in the intestinal crypt [7,23,56,110,111]. Akin to findings in the *Tas1r3^−/−^* mouse, the ETC hyperplasia observed after infection with *Tritrichomnas ranier* in wild-type mice was absent in *Sucnr1^−/−^* mice [56]: while clearly implicating succinate in the response, the source of the succinic acid/succinate was not defined and one possibility is that *T. ranier* evoked changes in the gut microbiome, increasing the bacterial production of succinate.

Tuft cells throughout the intestinal tract have enriched mRNA expression of the free fatty acid receptor 3 (FFAR3), which binds short chain fatty acids (SCFA) such as butyrate, acetate and propionate [23,56,112]. Infection with helminth parasites can alter the cecal and colonic microbiomes to promote the growth of microbial communities producing SCFAs [113,114], making SCFAs a potential surrogate marker used by ETCs to detect parasitic infection. This could be particularly relevant to helminths that seek to reside in the colon. While oral supplementation with butyrate failed to induce tuft cell hyperplasia in mice [56], the connection between SCFAs, FFAR3 activity and the ETC warrants further research.

### 4.3. Damage-Associated Mediators and Other Immune Mediators May Coordinate Tuft Cell Activation

Parasite induced tissue damage can result in the release of ATP. In the airways and olfactory regions, tuft cells sense ATP via the P2Y2 receptor, following exposure to *Alternaria alternaria* extracts, to release cysteinyl leukotrienes [115]. Cysteinyl leukotriene E4 induces tuft cell hyperplasia in murine trachea in an IL-25-dependent and STAT6 (signal transducer and activator of transcription)-independent manner; the latter is intriguing as it disputes a role for IL-13 or IL-4 in this hyperplasia, although these cytokines can use other signal transduction molecules such as PI-3K [108]. Whether ETCs respond in a similar manner to ATP or other endogenous damage-associated molecular patterns (DAMPS, e.g., high mobility group box 1 (HMGB1)) is unknown. Mast cells release IL-25 and IL-33 in response to *H. polygyrus*-induced release of ATP from epithelial cells [43] and mast cell-derived IL-33 induces ILC2 production of IL-13 [43]: both events have the potential to drive ETC hyperplasia as a response to infection with helminth parasites.

## 5. Tuft Cells and Other Gastrointestinal Disorders

While not extensive, a small number of studies have examined ETCs in irritable bowel syndrome (IBS), inflammatory bowel disease (IBD), and upon enteric viral infection, prompting us to ask questions about the implications of these findings in the context of enteric parasitic infections [13,116,117,118,119,120,121].

### 5.1. Enteric Tuft Cells and Post-Parasitic Infection Irritable Bowel Syndrome

*Giardia duodenalis* (a protozoan) and *T. spiralis* have been incriminated as causative agents of post-infectious irritable bowel syndrome (PI-IBS) in humans, rats, or mice [122,123,124]. Patients with IBS can harbor more *Blastocystis* and *Cryptosporidium* protozoan parasites [125,126]. Recently, colonic biopsies from diarrhea-dominant IBS patients revealed a higher percentage of DCLK1^+^ ETCs and increased secretion of IL-25 following in vitro culture [116]. However, these descriptive studies are associative and while it is reasonable to speculate that protozoan-evoked ETC activation and subsequent hyperplasia could be relevant to the etio-pathogenesis of IBS, cause-and-effect data in support of this postulate are required.

### 5.2. Parasites as Immunotherapy for Inflammatory Bowel Disease: The Role of Tuft Cells

In order to be successful (survive and reproduce), endo-parasites must undermine their host’s attempt to destroy and/or eradicate them. There are many examples of helminth-induced immunoregulatory cells and mediators that would dampen host anti-worm strategies. Helminth therapy is a novel approach that aims to exploit the immunomodulatory potential of infection with helminths to treat inflammatory diseases (reviewed by [127]). Thus, infection with *Hymenolepis diminuta* (rat cestode), *Schistosoma mansoni* (blood fluke/trematode parasite), and parasitic nematodes (*H. polygyrus, Necator americanus* (human hookworm), *T. spiralis*, and *Trichuris suis*) has been found to alleviate the severity of disease in murine models of colitis (which shares some features with human IBD) [128,129,130,131]. ETC frequency and IL-25 level were both found to be downregulated in human colonic biopsies and mouse models of colitis [13,117,118,119], suggesting that one benefit of helminth therapy would be the correction of this deficit. Succinate supplementation in the diet of TNF^Δ^^ARE/+^ mice, which spontaneously developed a Crohn’s disease-like ileitis, resulted in less injury and reduced inflammation [13]. Furthermore, CD4^+^ T cells obtained from IBD patients when treated with IL-25, showed decreased production of IFN-γ, TNFα, and IL-17 [117]. These studies imply a beneficial anti-colitic role for the activation of ETCs, yet succinate can have many effects and IL-25 production is not the sole purview of the tuft cell. Again, it is imperative to advance the field with the application of a range of methodologies and transgenic mice to determine whether the absence of ETC exaggerates colitis and/or the activation of ETCs can be therapeutic in IBD.

### 5.3. The Role of Tuft Cells in Coinfections: Parasites and Viruses

In contrast to the role in anti-helminth responses, ETCs may facilitate enteric viral infection. The murine norovirus isolate CR6 (MNV.CR6) [132] was shown to directly infect ETCs by binding to CD300lf, a tuft cell specific protein [120,133,134], suggesting that murine norovirus is tuft cell tropic. In accordance, the ETC hyperplasia that follows infection with parasitic helminths leads to an increased MNV.CR6 load [120,135]. Infection with *T. spiralis* resulted in increased ileal loads of MNV (strain CW3) and impaired MNV-specific CD4^+^ and CD8^+^ T cell responses that were STAT6-dependent [135]. Coinfecting mice with *H. polygyrus* and West Nile Virus (WNV), a mosquito-borne flavivirus, exacerbated viral disease, as determined by increased weight loss, increased viral RNA levels, impaired CD8^+^ T cell responses and decreased survival [136]. In contrast, ETC-deficient *Pou2f3^−/−^* mice fared better than coinfected wild-type mice [136]. Additionally, succinate supplementation in WNV-infected wild-type mice resulted in complete mortality, while succinate-treated *Pou2f3^−/−^* mice displayed a mortality rate similar to control WNV-infected wild-type mice [136]. Finally, infection with *H. polygyrus* increased the host’s susceptibility to other flaviviruses, namely Powassan and Zika virus [136]. Although there was no evidence that norovirus actively replicated in ChAT positive tuft cells in human intestinal biopsies (jejunum, ileum, and colon) [137], the role of ETCs in human enteric viral infections is yet to be explored. Understanding the dynamics of coinfections with parasites and viruses is of importance in considering the outcomes of such comorbidities in parasite-endemic populations of the world. Moreover, one wonders if ETCs represent a drug target in helminth-infections; would this render the individual susceptible to viral enteritis, and similarly would this be the case if ETC biology were to be manipulated to medically manage IBS or IBD?

## 6. Conclusions

From a relatively obscure beginning based on morphological descriptions, recent years have seen the enteric tuft cell take center-stage as a sensor of intestinal parasites—mainly helminths – and as an important component of the initiation sequence to mobilize an effective anti-parasite response. Relying heavily on a small number of parasitic nematode-mouse laboratory models, the paradigm has arisen that tuft cell sensing of the parasite (or possibly surrogate signals) leads to IL-25 production, which evokes IL-13 synthesis from ILC2s to drive Th2 immunity and increase tuft cell differentiation. While tuft cells may be important in the initiation or orchestration of Th2-dominated anti-worm responses, they are not an essential requirement as their absence results only in the slowing of worm expulsion, not the chronic establishment of worms. The field of tuft cell biology is in its infancy and there is little doubt that parasite-specific impacts on tuft cells will emerge. Concerted research efforts are needed to determine which parasite-derived molecules are sensed by the tuft cells, to identify tuft cell activation markers, and to extend our knowledge beyond the tuft cell-ILC2 circuit to determine precisely how tuft cells affect neighboring epithelial cells and immune, neuronal and stromal cells in the mucosal environment (Box 1). The majority of the helminth-rodent models that explore ETC-mediated anti-parasitic function elicit Th2 responses in their hosts. Research is needed to assess whether ETCs are involved in coordinating host responses in other parasitic models of infection that are not Th2-centric, such as *Entamoeba histolytica*, *Giardia*, and *Schistosoma mansoni*. Finally, knowledge of human enteric tuft cells is rudimentary, and while it is challenging to perform cause-and-effect studies, we underscore the value in pursing this line of investigation with the vision that the pharmacological manipulation of tuft cell activity could be of benefit in treating infection with helminth (and possibly protozoan) parasites and unrelated auto-inflammatory conditions that affect the bowel.

Box 1 **Outstanding questions in tuft cell participation in parasitic infection.**

**Basic Biology:**

- How universal and necessary is tuft cell involvement in the successful eradication of protozoan and helminth parasites, including those that induce a Th1- or Th17-centric response?
- Are anti-parasitic responses limited to or dominated by a particular subtype of ETC?
- What are suitable markers to identify activated tuft cells and what are the dynamics of activation, differentiation, and function of different ETC subtypes?
- At steady state, do tuft cells communicate with neighboring cells (e.g., goblet cells, immune cells, fibroblasts, neurons) and, if so, which molecules are involved? How is this communication altered during infection?
- How do ETC-derived neuronal factors contribute to the host anti-parasitic response?
- What role, if any, do tuft cells play in chronic parasitic infections?
- How important are tuft cells in concomitant immunity to other infections, and the regulation of auto-inflammatory disease?
- What are the roles of ETCs in acute versus post-infectious events during parasitic infections?
- Are there are sex-related differences in tuft cells: baseline numbers, subtypes, induceability, and response to infection?

**Translational Relevance**

- How representative of human tuft cells are murine tuft cells?
- Can knowledge of tuft cells be used to intervene in the course of parasitic infection in humans, companion animals, and domestic livestock?
- Can drugs be developed to activate tuft cells in an organ-specific manner to treat auto-inflammatory diseases?

## Abbreviations

ETC	enteric tuft cell
ISC	intestinal stem cell
IL	interleukin
POU2F3	POU Domain Class 2 Transcription Factor 3
Gfi1b	Growth Factor Independent 1b
ATOH1	Atonal homolog 1
SOX4	Sry-box containing factor 4
TRPM5	transient receptor potential cation channel subfamily M member 5
DCLK1	doublecortin-like kinase 1
TSLP	thymic stromal lymphopoietin
ILC2	type 2 innate lymphoid cells
CysLT	cysteinyl leukotriene
LRMP1	lymphoid-restricted membrane protein
ITPR2	Inositol 1,4,5-Trisphosphate Receptor Type 2
ACh	acetylcholine
AAM	alternatively activated macrophages
TGFβ	transforming growth factor β
ATM	ataxia telangiectasia mutated
COX-2	cyclooxygenase 2
ChAT	choline acetyltransferase
GABA	gamma-aminobutyric acid
5-HT	5-hydroxytryptamine
TPH-1	tryptophan hydroxylase
ESP	excretory/secretory products
GPCR	G-protein-coupled receptor
IEC	intestinal epithelial cells
SCFA	short-chain fatty acid
FFAR3	free fatty acid receptor 3
STAT	signal transducer and activator of transcription
DAMPS	damage-associated molecular patterns
HMGB1	high mobility group box 1
IBS	irritable bowel syndrome
IBD	inflammatory bowel disease
MNV	murine norovirus
WNV	West Nile Virus
